# Multidisciplinary Approach in Rare, Fulminant-Progressing, and Life-Threatening Facial Necrotizing Fasciitis

**DOI:** 10.3390/idr16060084

**Published:** 2024-11-01

**Authors:** Mihaela Pertea, Stefana Luca, Raluca Tatar, Bogdan Huzum, Mihai Ciofu, Vladimir Poroch, Dragos Octavian Palade, Daniela Vrinceanu, Mihail Balan, Oxana Madalina Grosu

**Affiliations:** 1 Department Plastic Surgery and Reconstructive, Faculty of Medicine, “Grigore T. Popa” University of Medicine and Pharmacy, 700115 Iasi, Romania; mihaela.pertea@umfiasi.ro (M.P.); oxana-madalina.grosu@umfiasi.ro (O.M.G.); 2 Department of Plastic Surgery and Reconstructive Microsurgery, “Sf. Spiridon” Emergency County Hospital, 700111 Iasi, Romania; 3 Faculty of Medicine, “Carol Davila” University of Medicine and Pharmacy, 020021 Bucharest, Romania; raluca.tatar@umfcd.ro; 4 Department of Plastic Reconstructive Surgery and Burns, “Grigore Alexandrescu” Clinical Emergency Hospital for Children, 011743 Bucharest, Romania; 5 Department of Surgery II, Faculty of Medicine, “Grigore T. Popa” University of Medicine and Pharmacy, 700115 Iasi, Romania; bogdan.huzum@umfiasi.ro (B.H.); octavian.palade@umfiasi.ro (D.O.P.); 6 Department of Orthopaedics and Traumatology, “Sf. Spiridon” Emergency County Hospital, 700111 Iasi, Romania; 7 Faculty of Dental Medicine, “Grigore T. Popa” University of Medicine and Pharmacy, 700115 Iasi, Romania; mihai.ciofu@umfiasi.ro (M.C.); mihail.balan@umfiasi.ro (M.B.); 8 Oro-Maxillo-Facial Clinic, “Sf. Spiridon” Emergency County Hospital, 700111 Iasi, Romania; 9 Department of Palliative Care, “Grigore T. Popa” University of Medicine and Pharmacy, 700115 Iasi, Romania; vladimir.poroch@umfiasi.ro; 10 Department of Palliative Care, Regional Institute of Oncology, 700483 Iasi, Romania; 11 ENT Clinic, “Sf. Spiridon” Emergency County Hospital, 700111 Iasi, Romania; 12 ENT Department, Emergency University Hospital, 050098 Bucharest, Romania; daniela.vrinceanu@umfcd.ro; 13 ENT Department, Carol Davila University of Medicine and Pharmacy, 020021 Bucharest, Romania

**Keywords:** facial trauma, necrotizing fasciitis, fulminant progression, debridation, reconstructions

## Abstract

(1) Background: Necrotizing fasciitis is known as a severe condition with a high risk of mortality, placing it among the most feared infections. In most cases, it has a polymicrobial etiology (type 1), requiring complex treatment that is continuously adapted to the evolving microbiological status. The facial localization of the disease is rare, fulminant progressing, and is often life-threatening. (2) Methods: We present the case of a patient with multiple comorbidities who, following trauma to the nasal dorsum, developed a wound with a rapid and severe progression to extensive bilateral periorbital necrosis. This was accompanied by a dramatic deterioration in their general condition, a polymicrobial biological status, and fluctuating progression despite instituted treatment (both medical and surgical). (3) Results: The patient required multiple surgical interventions by multidisciplinary teams (plastic surgery; ear, nose, and throat specialist (ENT); maxillofacial surgery; and ophthalmology), experiencing periods of a severe, life-threatening general condition, necessitating prolonged orotracheal intubation. Wounds with fluctuating progression, extensive skin necrosis, and significant post-excisional soft tissue defects required skin graft coverage. The result meant a saved life and functional and aesthetic sequelae at the level of the face. (4) Conclusions: Necrotizing fasciitis of the face is a rare and severe disease that must be recognized early and treated appropriately by a multidisciplinary team to save the patient’s life and minimize the resulting functional and aesthetic sequelae.

## 1. Introduction

Necrotizing fasciitis (NF) is a rare, very aggressive pathology with a fulminant progression, potentially causing death. The rarity of this condition is even greater when it is located on the face. Miller et al. in 2021 reported an annual incidence of 500–1000 cases. The facial localization is found in 5.3% of cases [1].

In most cases, facial NF has an odontogenic cause [1]. Despite this, reports indicate that the localization of necrotizing fasciitis (NF) in the lower limb is a negative prognostic sign. This can be explained by the rarity of facial localizations and the inability to achieve statistically significant assessments. The risk of NF can be evaluated using various severity scores. Among these, the most well known is the Laboratory Risk Indicator for Necrotizing Fasciitis (LRINEC) score, described by Wong et al. in 2004. This score considers serum values of C-reactive protein, white blood cells, hemoglobin, serum sodium, and serum glucose [2]. In 2019, Cribb et al. described the SIARI score to differentiate NF from other soft tissue infections. SIARI stands for Site other than the lower limb, Immunosuppression, Age < 60 years, Renal impairment (creatinine > 141 µmol/L), and Inflammatory markers (CRP ≥ 150 mg/dL, WCC > 25 × 10^6^ µ/L) [3]. To improve the sensitivity and specificity of these risk scores for NF, Breidung et al. in 2022 described the Laboratory and Anamnestic Risk Indicator for Necrotizing Fasciitis (LARINF) score. This score takes into account hemoglobin levels, C-reactive protein, procalcitonin, and the presence of three comorbidities [4]. The LRINEC score has a sensitivity of 70% and a specificity of 60%, whereas the LARINF score has a sensitivity of 84% and a specificity of 75% [5].

Most often, NF is determined by polymicrobial flora in 70–90% of cases [1,6]. The most frequently encountered pathogen is Streptococcus pyogenes [7,8]. From a microbiological point of view, NF is classified into four types: I—polymicrobial infection, II—monomicrobial infection, III—infection determined by Gram-negative pathogens, and IV—fungal infection [5]. Most often, patients present with erythema, fever, edema, tachycardia, tenderness to palpation, or crepitus, with a rapidly deteriorating general condition [1,9,10]. Miller et al., in a study conducted on a group of 209 patients, reported a mortality rate of 18% [5].

The treatment requires an aggressive approach. When the condition is localized to the facial region, it is essential to involve a multidisciplinary team, including specialists in otolaryngology (ENT), maxillofacial surgery, and plastic surgery, with continuous support from an intensive care unit [5]. Alongside these specialists, an infectious disease expert plays a significant role in determining the appropriate antibiotic treatment according to the identified microbial spectrum [11]. The treatment should address hemodynamic support, cover the entire microbial spectrum with antibiotic therapy, and, of course, include a surgical intervention focus [12]. Since NF is considered a surgical emergency, Ferzli et al. report the necessity of serial debridement, with an average of 4.6 surgical interventions per week [13]. The final stage of surgical treatment involves covering the remaining soft tissue defects with split-thickness skin grafts or various types of flaps, even skin substitutes [5,14,15,16,17].

Mortality in cervicofacial NF, as reported by Yan et al., is 12.3%, while Gunaratne et al. report it to be 13.4% [18].

We report the case of a 56-year-old patient diagnosed with non-odontogenic facial necrotizing fasciitis (NF), characterized by a fulminant and rapidly fluctuating course, involving polymicrobial flora, which posed a life-threatening risk. The patient’s critical condition necessitated intensive therapy, orotracheal intubation followed by tracheostomy, and multiple surgical interventions, all supported by targeted antibiotic treatment.

## 2. Case Report

### 2.1. Clinical Presentation

**Figure 1:**
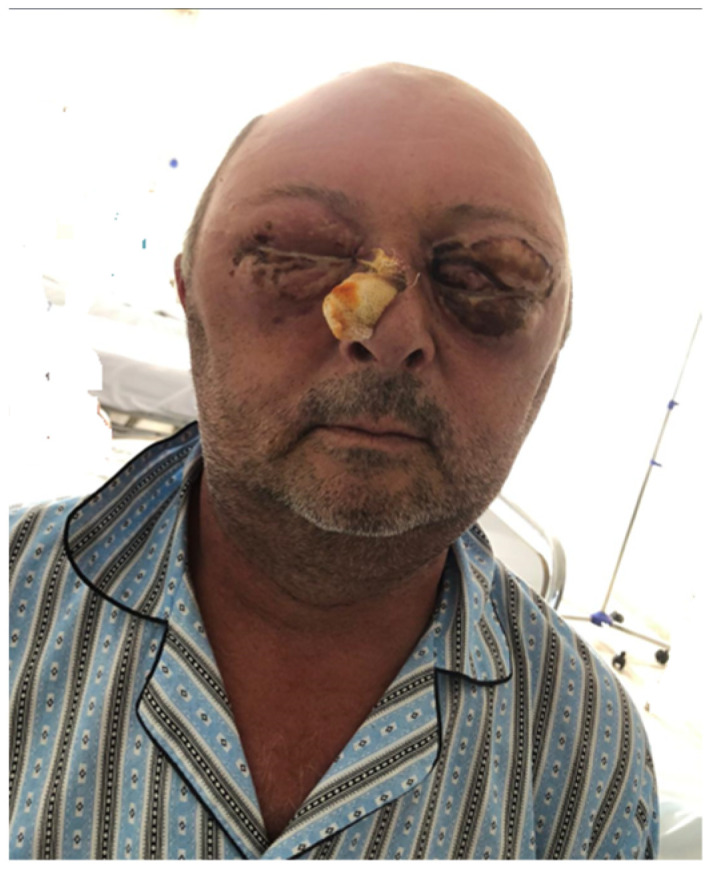
The clinical appearance of the patient at the time of admission to the hospital (areas of necrosis in the eyelids of both eyes).

A 56-year-old male patient with a history of chronic alcohol consumption presented to the ENT department with a traumatic brain injury and craniofacial trauma. He also had a contused and infected wound at the base of the nose on the dorsal side, sustained from a fall at ground level 48 h prior to presentation (Figure 1).

On clinical examination, there was significant periocular edema and erythema. The wound on the dorsal side of the nose measured 2.5 cm in length and 0.5 cm in width, with irregular margins and blood-crusted edges. The wound exhibited a viscous, yellow, purulent secretion that was odorless. The patient’s overall condition was compromised.

### 2.2. Biological Markers

The biological constants at admission showed a WBC of 17.9 (1000 cells/µL), fibrinogen at 796 mg/dL, and CRP at 19.63 mg/L (Table 1).

Considering the values of these biological constants, the following scores were calculated: the LRINEC score—over 8 (high), SIARI score, and LARINF score. They indicated the severity of the disease and the risk of NF.

**Figure 2:**
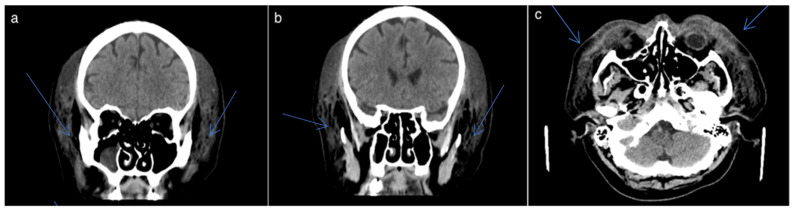
(**a**–**c**) Hematomas were also observed in the soft tissues of the face, including the perizygomatic areas bilaterally, anterior to the maxillary sinuses bilaterally, peri- and latero-orbital regions bilaterally, and perinasal areas, with a maximum thickness of 35 mm with significant swelling.

The radiologic examination (AP and lateral views) revealed a fracture of the nasal pyramid. Primary surgical cleaning of the wound on the nose’s dorsum was performed after collecting secretions from this site to identify the pathogen and perform an antibiogram to guide targeted antibiotic treatment. The CT scan showed large epicranial hematomas arranged in a circular pattern around the cranial vault, involving the frontal region bilaterally and to a lesser extent the occipital region, with a maximum thickness of 31 mm, along with subcutaneous emphysema. Hematomas were also observed in the soft tissues of the face, including the perizygomatic regions bilaterally, the areas anterior to the maxillary sinuses bilaterally, the peri- and latero-orbital regions bilaterally, and the perinasal areas, with a maximum thickness of 35 mm. A subdural lining was observed in the right temporal area, posterior to the Sylvian fissure, with a maximum thickness of 2–2.5 mm, which is difficult to distinguish from the adjacent parenchymal density. Correlation with clinical symptoms is necessary, with potential re-evaluation if symptoms worsen. A discontinuity and slight angulation were also noted in the small bones of the nose on the left side in the anterior portion (Figure 2).

**Figure 3:**
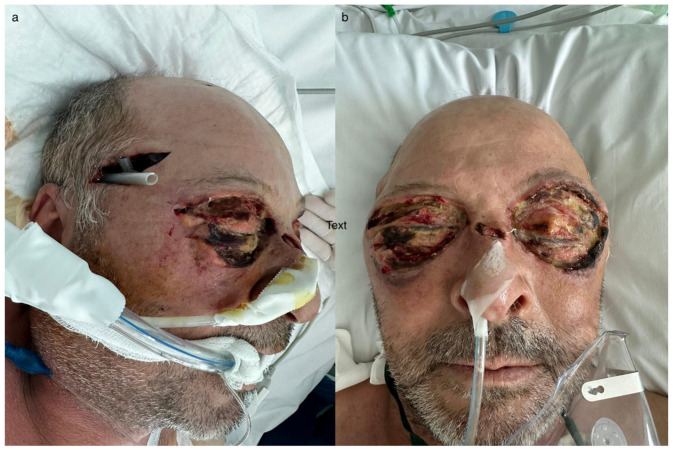
Postoperative view. (**a**) Temporal skin incisions. (**b**) Aspect after first necrectomy. (**a**,**b**) Extensive areas of necrosis around both eyes.

Based on the clinical presentation, biological markers, imaging findings, and risk score assessments, an emergency surgical intervention was indicated under general anesthesia with orotracheal intubation (OTI). A combined maxillofacial and ENT surgical team performed two temporal skin incisions, approximately 3–4 cm in length, extending down to the superficial fascia of the temporal muscle, allowing for the evacuation of purulent secretions. Additionally, two intraoral incisions were made at the level of the upper buccal vestibule, extending to the retrotuberosity. These two incisions were connected via a drainage tube that exited the skin (Figure 3).

Necrectomy was also carried out on the wound on the back of the nose. Antibiotic treatment with Penicillin G at 4 million units every 6 h and Clindamycin at 600 mg every 8 h (treatment recommended by the infectious disease specialist) was initiated until microbiological results and the antibiogram were obtained.

**Figure 4:**
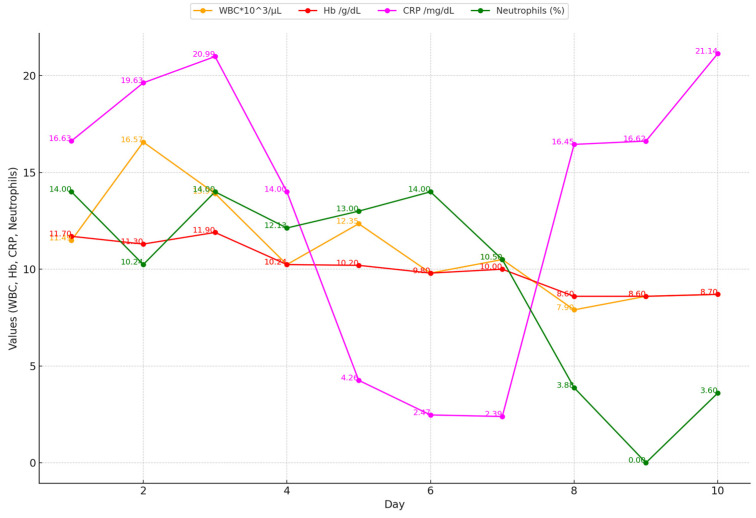
The progression of biological constants in the first 10 days after hospital admission. WBCs—white blood cells; Hb—hemoglobin; CRP—C-reactive protein.

The patient’s general condition deteriorated, with necrotic areas on the face rapidly expanding, accompanied by a decline in biological markers (Figure 4).

### 2.3. Microbiological Findings

*Streptococcus pyogenes—naturally sensitive to penicillin*—was initially detected in the secretions (during the first day). Each surgery was carried out by a mixed team of plastic and maxillofacial surgeons. On the 7th day, the presence of *Acinetobacter baumannii* was confirmed. On the 10th day, *Klebsiella pneumoniae* was detected, and on the 12th day, *Pseudomonas aeruginosa* was identified, indicating a polymicrobial status. Based on the results of the antibiogram and the guidance of the infectious disease specialist, the treatment regimen was adjusted. The patient was treated with Meropenem at a dose of 3 g/day for 7 days and Colistin at 4.5 mg twice a day for 15 days.

Until the secretions were negative on the 21st day, only the *Pseudomonas aeruginosa* infection persisted in the last 4 days. On the 11th day, *Candida* spp. were detected in the tracheal aspirate (the patient being intubated via an orotracheal tube), and on the same day, *Klebsiella pneumoniae* was found in the urine culture.

### 2.4. Surgical Interventions

**Figure 5:**
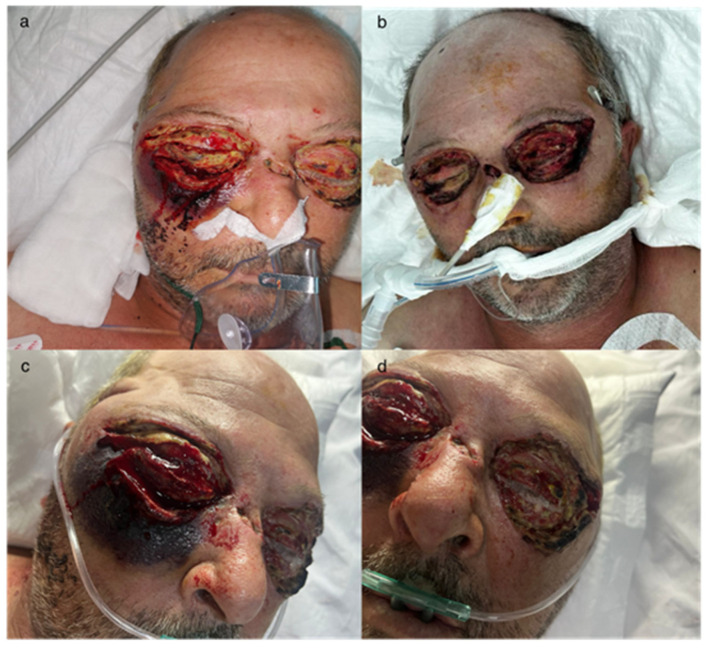
The extension of the areas of necrosis after the first necrectomy with the worsening of the general condition. (**a**) Day 4 after the first excision, (**b**–**d**) unfavorable progression with the extension of necrotic areas.

The patient became hemodynamically unstable and was orotracheally intubated on the third day of hospitalization due to the fulminant expansion of necrotic areas despite the local and general treatment that had been initiated (Figure 5).

**Figure 6:**
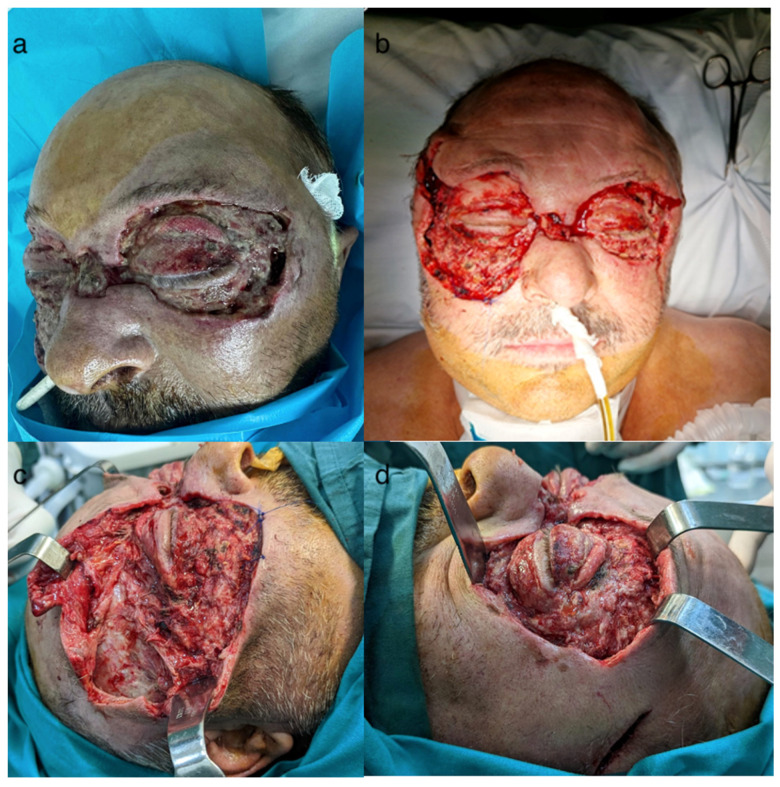
Post-excisional appearance: (**a**) first day after last excision, (**b**–**d**) 5th day post-excision—favorable progression.

**Figure 7:**
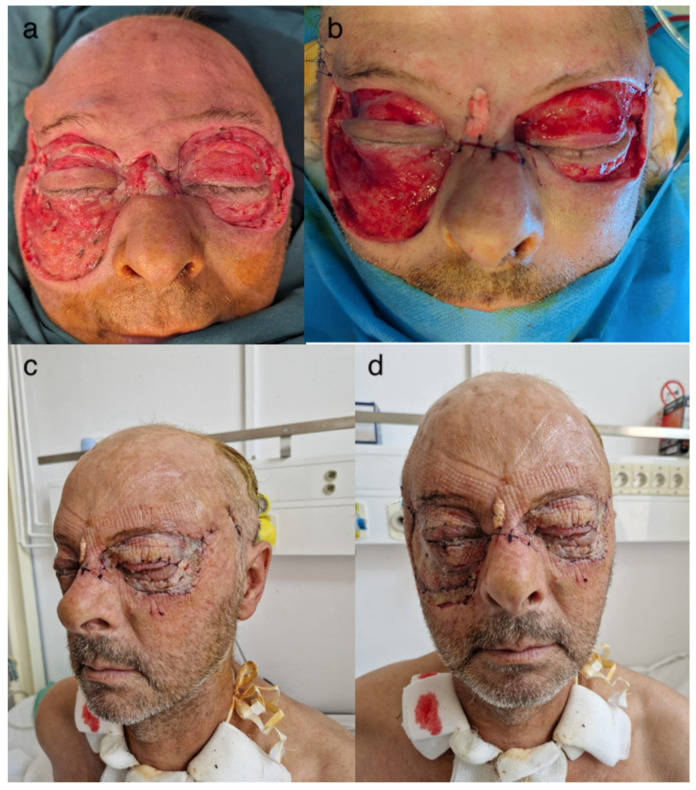
(**a**,**b**) Granulating wounds; (**c**,**d**) split-thickness skin graft, right lateral and left lateral views, respectively.

Repeated surgical interventions were performed for extensive necrectomy in the periocular, nasal dorsum, and zygomatic regions (Figure 6 and Figure 7). The patient’s general condition continued to deteriorate, with inflammatory markers showing a fluctuating course despite all treatments.

Antibiotic treatment was managed by the infectious disease specialist with targeted antibiotics for each detected pathogen. Dressings were changed daily, and between days 5 and 10, they were changed twice a day, resulting in a favorable local status (Figure 6).

### 2.5. Outcome

Due to the prolonged period of intubation (over 2 weeks) and the necessity of multiple serial surgical interventions, the patient underwent tracheostomy, which was maintained until the final surgical procedure involving split-thickness skin grafting. Skin grafts were harvested from the inner right arm. The placement of the skin grafts at the defect site was performed with careful attention to the aesthetic units of the face. These considerations were made to optimize both functional and, importantly, aesthetic outcomes as much as possible.

Negative pressure therapy was attempted, keeping it at a very low pressure, but the patient’s psychomotor agitation allowed it to be maintained for only a few hours. Daily dressings led to the formation of granular areas, which later allowed for coverage with split-thickness skin grafts (Figure 7).

Histological examination from the orbicularis muscle specimen, left orbital fat, and left cheek revealed extensive areas of necrosis associated with acute polymorphic inflammation, fibrin bands, and young granulation tissue. The necrotic areas involved adipose tissue, loose connective tissue, and striated muscle, with ulceration in some fragments. Marked congestion with recent thrombotic events, some with obliterative characteristics; foci of vasculitis; and polymorphic perivascular inflammation were also observed. All identified morphological features are compatible with necrotizing fasciitis.

**Figure 8:**
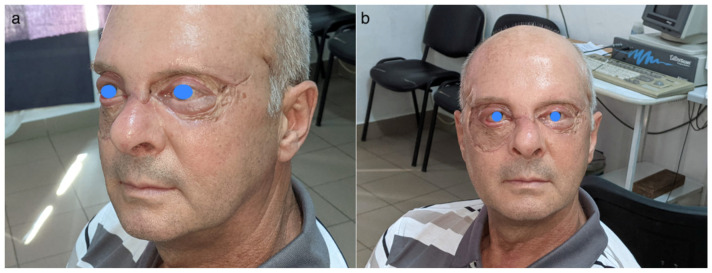
Appearance 3 months after discharge from hospital.

The patient’s recovery progressed well, leading to discharge after 40 days of hospitalization. Two months after discharge, the patient was readmitted for surgery to correct right eye eyelid insufficiency (Figure 8).

## 3. Discussion

Necrotizing fasciitis (NF) is undoubtedly a medical–surgical emergency. It involves the infection of the skin and soft tissues down to the deep fascia, leading to a severe systemic infection [1]. NF frequently localizes in the lower limbs. Pertea et al., in 2023, reported a systematic review that includes 34 cases of NF affecting the face documented in the literature [14]. The reported mortality for this condition ranges between 12% and 28% [5]. Agrawal Srikant reports a broad range of mortality rates for NF, spanning from 8.3% to 73% [19]. To emphasize the severity of this disease, various biological parameters have been analyzed and defined over time, contributing to the diagnosis and risk assessment of NF (such as LRINEC, SIARI, and LARINF scores) [2,3]. Additionally, the POSSUM score (Physiological and Operative Severity Score for the Enumeration of Mortality and Morbidity) is used for various conditions [20,21].

Recognizing the signs and symptoms, understanding the risk factors, and accurately interpreting imaging findings are crucial for an early diagnosis and treatment, which significantly reduces the risk of mortality. The facial localization of necrotizing fasciitis (NF) is rare, particularly when not associated with an odontogenic origin. The disease’s signs and symptoms are not pathognomonic, but the presence of erythema, edema, pain, and necrotic areas—especially in a traumatic context and in conjunction with a deteriorating general condition in a patient with multiple comorbidities—can strongly suggest a diagnosis of NF. Megas et al. report a significant association between ICU admission and mortality in NF patients, with approximately 25% of those admitted to the ICU for NF succumbing to the disease [5]. The reported case, despite a very high risk of death, resulted in the patient being discharged in a good general condition. This patient had an extended stay in the ICU, was orotracheally intubated, and then tracheostomized for approximately three weeks, and had a total hospitalization period of 40 days. Ultimately, the patient was discharged with integrated grafts and healed wounds. Notably, in this case, the microbial flora were diverse, initially detecting *Streptococcus pyogenes* and *Staphylococcus aureus*, followed by *Acinetobacter baumannii* on the 7th day, and *Klebsiella pneumoniae* by the 10th day, with a continuous adaptation of the antibiotic treatment. Lee et al. reported in 2017 a similar case but in which only *Pseudomonas aeruginosa* was detected, the patient having many comorbidities (hypertension, hepatitis C, chronic kidney disease). Also, Pertea et al. reported in 2023 a case of necrotizing fasciitis on the face but with localization on one hemiface, with eye damage, which required exenteration and in which a polymicrobial infection was detected [14].

Consistent with the literature, the most commonly involved pathogens in such cases include beta-hemolytic *Streptococcus*, *Staphylococcus epidermidis*, *Pseudomonas aeruginosa*, the *Streptococcus milleri* group, *Acinetobacter* spp., and *Enterobacter cloacae* [18].

The case described demonstrates a severe and rapid progression of the disease in a patient in their 50s, with a history of alcohol use but no associated comorbidities, who sustained trauma. While serum levels of C-reactive protein (CRP) and fibrinogen showed a downward trend with the administered antibiotic treatment, the white blood cell count peaked on the 10th day. Surgical treatment for necrotizing fasciitis of the face involves extensive excisions, typically performed in multiple stages. The literature recommends these procedures at intervals ranging from 2 to 4 days [22,23]. Residual soft tissue defects can be reconstructed using various surgical techniques, from full-thickness grafts to local or distant flaps [24]. In the reported case, due to the localization and significant extent of necrotic areas and subsequent defect sizes, only split-thickness skin grafts were utilized. To improve aesthetic outcomes, the placement of skin grafts adhered to the facial aesthetic units [14,24]. However, complications such as contractile scars affecting eyelid function can occur during the healing process.

As with many cases reported in the literature, the role of the multidisciplinary team was crucial in this case, involving plastic surgeons, maxillofacial surgeons, ENT specialists, ophthalmologists, intensivists, and infectious disease specialists [25]. Although relatively atypical, the use of biological markers at the time of hospital admission and the calculation of risk scores facilitated an early diagnosis and the rapid initiation of medical treatment, followed by emergency surgery and subsequent operations, ultimately saving the patient’s life.

## 4. Conclusions

Due to the still high mortality rate of NF, it is imperative to know the signs and symptoms, possible locations and etiological contexts of the disease, potential microbial agents involved, and risk factors. Additionally, knowledge of biological constants and their values that can guide diagnoses is essential in establishing risk scores. The necessity of “aggressive surgery” with extensive debridement combined with targeted antibiotic therapy involves a crucial step towards a favorable outcome and saving the patient’s life. The complexity and gravity of these cases of necrotizing fasciitis on the face, in most cases, require a long-term treatment, in several stages and always with the involvement of a multidisciplinary medical team.

## Figures and Tables

**Table 1:** Biological constants at admission.

Biological Constants at Admission	
WBC (1000 cells/µL)	17.9
C-reactive protein (mg/L)	19.63
Hemoglobin (g/dL)	10
Sodium (mmol/L)	142
Creatinine (mg/dL)	0.55
Glucose (mg/dL)	112

## Data Availability

The original contributions presented in the study are included in the article, further inquiries can be directed to the corresponding authors. The data can be obtain from the corresponding author upon resonal request. The original contributions presented in this study are included in the article.
